# Origin and Early Diversification of the Papain Family of Cysteine Peptidases

**DOI:** 10.3390/ijms241411761

**Published:** 2023-07-21

**Authors:** Dušan Kordiš, Vito Turk

**Affiliations:** 1 Department of Molecular and Biomedical Sciences, J. Stefan Institute, 1000 Ljubljana, Slovenia; 2 Department of Biochemistry, Molecular and Structural Biology, J. Stefan Institute, 1000 Ljubljana, Slovenia; 3 Jožef Stefan International Postgraduate School, Jamova 39, 1000 Ljubljana, Slovenia

**Keywords:** papain family, cysteine peptidases, phylogenomic analysis, evolution

## Abstract

Peptidases of the papain family play a key role in protein degradation, regulated proteolysis, and the host–pathogen arms race. Although the papain family has been the subject of many studies, knowledge about its diversity, origin, and evolution in Eukaryota, Bacteria, and Archaea is limited; thus, we aimed to address these long-standing knowledge gaps. We traced the origin and expansion of the papain family with a phylogenomic analysis, using sequence data from numerous prokaryotic and eukaryotic proteomes, transcriptomes, and genomes. We identified the full complement of the papain family in all prokaryotic and eukaryotic lineages. Analysis of the papain family provided strong evidence for its early diversification in the ancestor of eukaryotes. We found that the papain family has undergone complex and dynamic evolution through numerous gene duplications, which produced eight eukaryotic ancestral paralogous C1A lineages during eukaryogenesis. Different evolutionary forces operated on C1A peptidases, including gene duplication, horizontal gene transfer, and gene loss. This study challenges the current understanding of the origin and evolution of the papain family and provides valuable insights into their early diversification. The findings of this comprehensive study provide guidelines for future structural and functional studies of the papain family.

## 1. Introduction

The papain family (peptidase C1A family in InterPro (IPR013128), subfamily C1A peptidases in the MEROPS database (Db)) is the largest and best-characterised group of cysteine peptidases, named after the first archetype, the plant cysteine protease papain. Members of this family are widely distributed in Archaea, Bacteria, Eukaryota, and some viruses [1,2]. Papain-like peptidases are involved in numerous physiological and pathological processes, parasitic infections, and host defence. In parasitic protozoa, C1A peptidases participate in diverse processes, such as host cell and tissue invasion, encystation/excystation, catabolism of host proteins, and both the stimulation and evasion of host immune responses [3]. In plants, C1A peptidases are involved in the stress response, mobilisation of storage proteins during seed germination, induction of defence reactions, senescence, and regulation of cell death [4,5,6]. They are central hubs in plant immunity and are required for their resistance to various pathogens. At the same time, C1A peptidases are targeted by secreted pathogen effectors to suppress immune responses. Consequently, they are subject to a coevolutionary host–pathogen arms race [5]. The most studied mammalian C1A peptidases are human lysosomal cysteine cathepsins, which are essential for antigen processing [7], ageing, neurodegeneration [8], cancer [9,10], cardiovascular diseases [11], signalling [12,13], cell death [14], and inherited diseases [15]. Their activity can be regulated by gene expression, post-translational modifications, activation of inactive zymogens, accessibility to cleave peptide bonds, compartmentalisation, metal binding, and endogenous and exogenous inhibitors [16]. Dysregulation of C1A peptidase expression, localisation, and proteolytic activity can disrupt cellular homeostasis.

The first crystal structure of the papain family that was determined is papain from papaya (*Carica papaya*) [17]. The crystal structures of diverse human lysosomal cysteine cathepsins were determined during the 1990s; the first was cathepsin B [18], followed by other cathepsins including K [19], L [20,21], H [22], X [23], V [24], C [25], S [26], and F [27]. For example, cathepsin B has an additional loop in the R-domain in addition to the well-known occluding loop [18], whereas cathepsin X has a loop that enables dimerization [28]. Noteworthy, the monomeric and dimeric forms of cathepsin X are active [28], whereas, for cathepsin C, only its tetrameric form is active [25]. The insertions of novel structural elements into the papain fold restrict access of the substrate to the active site at either side of the active site cleft. To date, more than 40 crystal structures of diverse papain family representatives have been determined [1]. The papain fold is composed of two domains: the left L-domain, which contains three α-helices, and the right R-domain, which contains a twisted β-sheet and two helices. The two domains are linked to each other, forming a deep active site cleft that acts as a substrate-binding groove, in which Cys25 is positioned at the N-terminus (left domain) and His159 is positioned in the R-domain. Both residues form an ion pair. The binding sites between the substrate and enzyme are the S2, S1, and S1′ sites [29]. Moreover, a combined approach including proteomics data and a structural analysis enabled the recognition of crucial regions and residues within cysteine cathepsins, which endow them with specific properties, thus highlighting an interplay between structural rigidity and flexibility on selectivity [30]. Very recently, a clustering of papain-like cysteine proteinases based on profile–profile mappings has shown structural conservation in addition to a well-preserved catalytic triad and oxyanion hole [31].

All cathepsins are monomers of approximately 30 kDa, with the exception of tetrameric cathepsin C [25,32] and dimeric cathepsin X [33]. Cathepsins differ in their specificity and tissue distribution [33]. Most cathepsins exhibit endopeptidase activity, whereas caths B, H, C, and X are the only known exopeptidases. However, cathepsins C and X are strict exopeptidases. Lysosomal cathepsins are synthesised as inactive zymogens. They are composed of propeptides that unfold at an acidic pH, thereby opening the active site of the enzyme [34]. Cathepsins are activated by autocatalytic processing [35,36,37] and by other proteases such as cathepsin C [38]. Equally important are cystatins, the cathepsins’ endogenous proteinase inhibitors, which are the most investigated. Cystatins are divided into three families: stefins, cystatins, and kininogens [39,40]. Kininogens are composed of two inhibitory cystatin-like domains. They are divided into low-molecular-weight (LK) and high-molecular-weight (HK) kininogens. Both LK and HK kininogens bind to two molecules of cathepsins with high affinity, which is unique among cathepsins [41,42]. More information about the structure–function relationships of cystatins and the origin and evolution of the cystatin superfamily can be found in [43,44].

The evolutionary analyses of the papain family started in the early 1990s, during the pre-genomic era [45,46,47], and were based on a small sample of organisms and the limited diversity within the papain family that was available at that time. Since then, the number of representatives of the papain family has increased significantly, largely due to the accumulation of eukaryotic and prokaryotic transcriptomic and genomic sequences. In this study, we aimed to obtain a comprehensive insight into the distribution, origin, and early diversification of the papain family in Eukaryota, Bacteria, and Archaea. Such an analysis could not have been previously performed because of the limited number of available eukaryotic and prokaryotic genomes. We used significantly expanded taxon sampling compared to previous studies, in which, for the first time, all major eukaryotic and prokaryotic lineages were represented [48,49]. In particular, we included data from previously unsampled eukaryotic lineages to represent all eukaryotic supergroups [48]. We traced the birth and expansion of the papain family with a phylogenomic analysis, using publicly available information from numerous prokaryotic and eukaryotic proteomes, transcriptomes, and genomes. We found that the papain family expanded greatly during eukaryogenesis through massive gene innovation and diversification, which resulted in eight ancestral C1A lineages in the ancestor of eukaryotes. The papain family expanded further during eukaryotic evolution, especially through extensive gene duplications in the ancestral cathepsin L and B lineages. Together, we demonstrated that diversification of the papain family predates the origin of eukaryotes and that a burst of innovation during eukaryogenesis led to a eukaryotic ancestor with a complex set of ancestral C1A lineages.

## 2. Results and Discussion

### 2.1. The Papain Family Is Highly Represented in Omics Databases

Notably, the number of C1A peptidases in the proteomic, transcriptomic, and genomic databases is significantly different. Proteomic databases have the lowest numbers because many organisms lack proteomic data, but unannotated data exist for these organisms in transcriptomic or genomic databases. Although the papain family is well represented in the MEROPS database (release 12.4) with >17,000 sequences [1], much higher numbers of members of the papain family are present in general proteomic databases, such as InterPro and Superfamily. More than 60,000 C1A peptidases are present in InterPro database (IPR000668—Peptidase C1A, papain C-terminal; IPR038765—Papain-like cysteine peptidase family); however, vast numbers are present in transcriptomic and genomic databases. In this study, we obtained unbiased insights into the complete repertoire of the papain family across all major eukaryotic and prokaryotic lineages. A phylogenomic approach was used to analyse the papain family, in which we placed the proteome/transcriptome/genome data into an evolutionary context. To find new members of the papain family, we searched all publicly available proteomic, transcriptomic, and genomic databases of key eukaryotic taxa and/or lineages. We limited the search in the National Center for Biotechnology Information (NCBI) TSA transcriptomic and WGS genomic databases to specific taxonomic groups. Searching for C1A peptidases in the EukProt V3 transcriptomic database [50] was crucial because we obtained data for eukaryotic organisms and lineages that were not available in the NCBI Db. This approach was especially important for identifying new representatives of C1A orthologous gene families in a large number of diverse unicellular eukaryotes. Prokaryotes have only a small number of representatives of the papain family per species.

### 2.2. Early Diversification of the Papain Family in the Ancestor of Eukaryotes

**Figure 1:**
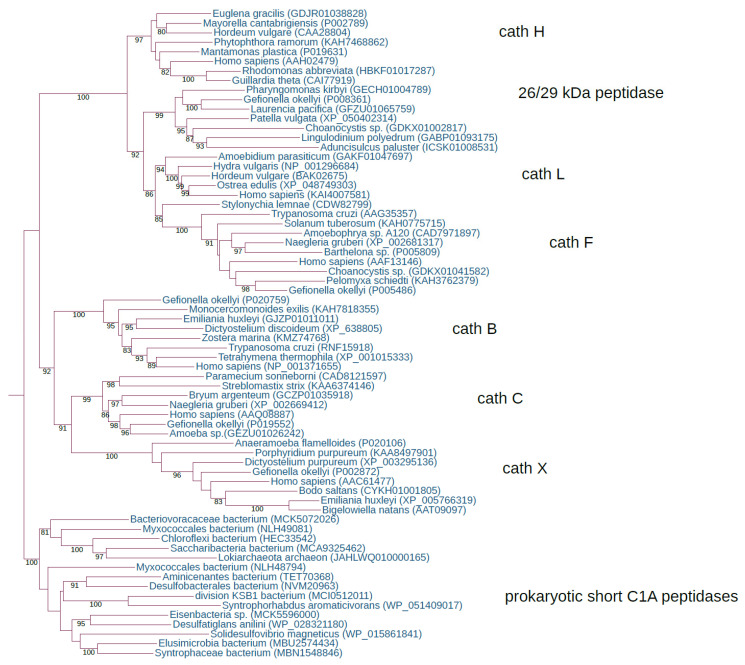
Early diversification of the papain family in the eukaryotic ancestor. The rooted ML tree shows the evolutionary relationships between the seven ancestral eukaryotic orthologous gene families, cathepsins B, C, X, L, H, and F, and the 26/29 kDa peptidase. The best-fit model, according to the Bayesian information criterion, was WAG + I + G4. The ML tree represents bootstrap consensus following 1000 replicates. Sequences were obtained from GenBank, and species names and accession numbers are included.

**Figure 2:**
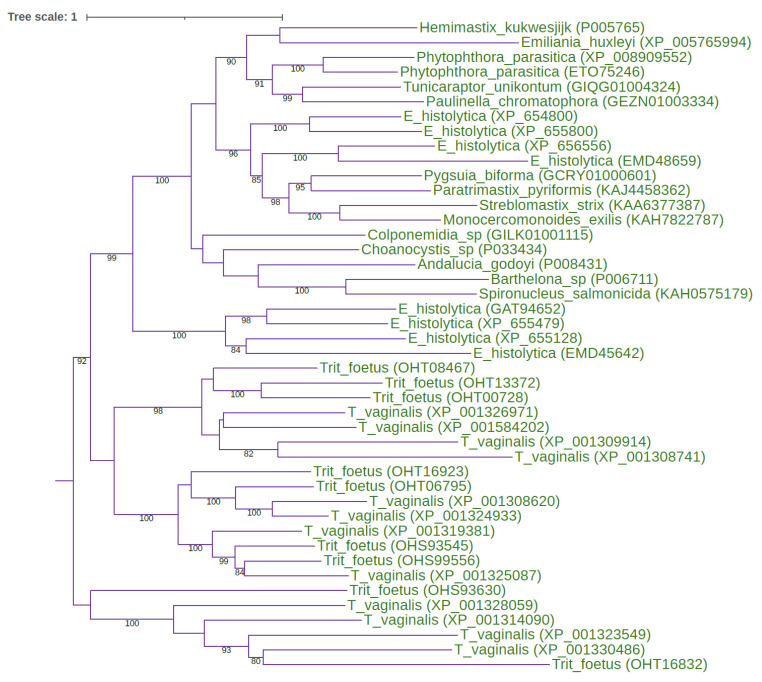
ML tree for the eukaryote-specific type 1 long C1 peptidases. The midpoint-rooted ML tree shows the evolutionary relationships inside the eukaryote-specific type 1 long C1 peptidases. The best-fit model, according to the Bayesian information criterion, was PMB + F + I + G4. The ML tree represents the bootstrap consensus following 1000 replicates. Sequences were obtained from GenBank, and species names and accession numbers are included. Abbreviations: T_vaginalis: *Trichomonas vaginalis*; Trit_foetus: *Tritrichomonas foetus*; E_histolytica: *Entamoeba histolytica*.

Using diverse eukaryotic representatives of the papain family (e.g., human, invertebrate, plant, and protist C1A sequences) as queries for searching eukaryotic proteome, transcriptome, and genome databases, we recognised the conserved repertoire of the eight ancestral C1A lineages that are present in all eukaryotic supergroups (Table 1 and Table 2). This distribution pattern demonstrated that eight ancestral eukaryotic paralogous C1A lineages were present in the last eukaryotic common ancestor (LECA). The eight ancestral eukaryotic paralogous C1A lineages are as follows: cathepsins B, C, X, L, H, F, 26/29 kDa peptidase, and type 1 long C1 peptidase. The maximum likelihood (ML) phylogenetic analysis of the papain family in key eukaryotic lineages provided strong evidence for the early diversification of the papain family (Figure 1 and Figure 2). 

Because most of these C1A lineages originated in the LECA by gene duplication, they are referred to as ancestral or ancient eukaryotic paralogs; some other C1As may have originated through horizontal gene transfer (HGT) from bacteria, which are referred to as ancestral eukaryotic pseudoparalogs [51]. Thus, the phylogenomic analysis of the papain family provided strong evidence of its origin (i.e., where and when the lineages originated, and from which progenitor). In early evolutionary studies of the papain family, there was much speculation concerning the nature of their ancestor [46,47], and only two classes, namely, cathepsins B and L, were recognised [45]. However, our exhaustive analysis of C1A peptidases showed that this widely accepted scheme for the evolution of the papain family [45] is insufficient and needs to be revised. Due to either bias and/or a limited number of C1A peptidase representatives, the papain family can neither be adequately classified nor mapped to the origin of its numerous orthologous gene families. In contrast to eukaryotes, no widespread orthologous gene families exist in Bacteria and Archaea. Moreover, using a diverse set of prokaryotic representatives of the papain family as queries for searching eukaryotic omics databases, we uncovered another hidden repertoire of short and long eukaryotic C1A peptidases. These C1A peptidases were horizontally acquired from bacteria independently several times into a few eukaryotic lineages, such as rotifers, green algae, stramenopiles, and fungi, which will be briefly described later. 

### 2.3. Distribution of the Papain Family in Eukaryotes and Prokaryotes

The phyletic distribution pattern of the papain family was analysed, which revealed the presence of this family in Bacteria, Archaea, and Eukaryota (Table 1, Table 2 and Table 3). 

#### 2.3.1. Papain Family in Eukaryotes

The phylogenomic analysis indicated a widespread distribution of the papain family in eukaryotes. However, the distribution patterns of the eight ancestral eukaryotic paralogous C1A lineages in eukaryotes are different (Table 1). Cathepsins B, C, X, and L are widespread throughout eukaryotes (Table 1), with only a few losses in Opisthokonta. Cathepsins B, C, X, and L are lost in Fungi and Rotosphaerida, whereas cathepsins C and X are also lost in Apusozoa (Table 2). Cathepsin F is similarly widespread, with few losses in Picozoa, Pluriformea, Fungi, and Rotosphaerida (Table 1 and Table 2). Cathepsin H is also present in major eukaryotic lineages but shows more losses in Picozoa, Hemimastigophora, Telonemia, Ancyromonadida, Malawimonadida, and Metamonada. However, in Obazoa, there is a remarkable number of losses in opisthokonts, namely, Choanoflagellata, Filasterea, Tunicaraptor, Ichthyosporea, Fungi, Rotosphaerida, and Breviatea (Table 1 and Table 2). The 26/29 kDa peptidase is widespread in eukaryotes, with only a few losses in Ancoracysta, Telonemia, and CRuMs, while in opisthokonts, few losses can be seen in Fungi, Rotosphaerida, Filasterea, and Tunicaraptor (Table 1 and Table 2). The 26/29 kDa peptidase is widespread in Metazoa, with a loss in the ancestor of Theria (marsupials and placental mammals). It is still present in monotremes (platypus + echidna) but absent in all other mammalian species. Type 1 C1 long peptidase (500–650 aa long) is a novel eukaryote-specific C1A peptidase with an unusual distribution in eukaryotes since it is lost in multicellular lineages (animals and plants) (Table 1 and Table 2). Although this orthologous gene family is present in all three major eukaryotic domains (Diaphoretickes, Amorphea, and “Excavata”), there were a number of losses in Archaeplastida (plants and relatives), Picozoa, Cryptista, Ancoracysta, Telonemia, CRuMs, Ancyromonadida, and Malawimonadida. In opisthokonts, this C1A peptidase is present only in Tunicaraptor and is absent in metazoans, Fungi, choanoflagellates, Filasterea, Pluriformea, Ichthyosporea, and Rotosphaerida. It is present in Breviatea but lost in Apusozoa; these are two sister lineages of opisthokonts and are representatives of the Obazoa domain (Table 2). The largest number of representatives of this novel C1A peptidase is found in the SAR (Stramenopiles, Alveolata and Rhizaria) supergroup (especially in oomycetes), Haptophyta, Amoebozoa, and Metamonada.

**Table 1:** Distribution of the eight ancestral eukaryotic C1A paralogous lineages in Eukaryota.

	Cath B	Cath C	Cath X	Cath L	Cath F	Cath H	26/29 kDa Peptidase	Type 1 C1 Long
**Diaphoretickes**	■	■	■	■	■	■	■	■
Chloroplastida	■	■	■	■	■	■	■	□
Glaucophyta	■	■	■	■	■	■	■	□
Rhodophyta	■	■	■	■	■	■	■	□
Picozoa	■	■	■	■	□	□	■	□
Cryptista	■	■	■	■	■	■	■	□
Haptophyta	■	■	■	■	■	■	■	■
Centroheliozoa	■	■	■	■	■	■	■	■
Provora	■	■	■	■	■	■	□	□
Hemimastigophora	■	■	■	■	■	□	■	■
Telonemia	■	■	■	■	■	□	□	□
Stramenopiles	■	■	■	■	■	■	■	■
Alveolata	■	■	■	■	■	■	■	■
Rhizaria	■	■	■	■	■	■	■	■
**Amorphea**	■	■	■	■	■	■	■	■
Amoebozoa	■	■	■	■	■	■	■	■
Obazoa	■	■	■	■	■	■	■	■
CRuMs	■	■	■	■	■	■	□	□
Ancyromonadida	■	■	■	■	■	□	■	□
Malawimonadida	■	■	■	■	■	□	■	□
**“Excavata”**	■	■	■	■	■	■	■	■
Discoba	■	■	■	■	■	■	■	■
Metamonada	■	■	■	■	■	□	■	■

Black square represents presence, while white square represents absence.

**Table 2:** Distribution of the eight ancestral eukaryotic C1A paralogous lineages and two evolutionary younger C1A lineages in Obazoa.

	Cath B	Cath C	Cath X	Cath L	Cath F	Cath H	Cath O	26/29 kDa Peptidase	Type 1 C1 Long	VWFA-C1
**Opisthokonta**	■	■	■	■	■	■	■	■	■	■
Metazoa	■	■	■	■	■	■	■	■	□	■
Choanoflagellata	■	■	■	■	■	□	■	■	□	■
Filasterea	■	■	■	■	■	□	■	□	□	□
Tunicaraptor	■		■	■	■	□	■	□	■	□
Pluriformea	■	■	■	■	□	■	■	■	□	□
Ichthyosporea	■	■	■	■	■	□	■	■	□	□
Rotosphaerida	□	□	□	□	□	□	□	□	□	□
Fungi	□	□	□	□	□	□	□	□	□	■
**Breviatea**	■	■	■	■	■	□	□	■	■	□
**Apusozoa**	■	□	□	■	■	■	■	■	□	□
**Metazoa**										
Basal metazoans	■	■	■	■	■	■	■	■	□	■
Bilateria	■	■	■	■	■	■	■	■	□	■
Protostomia	■	■	■	■	■	■	■	■	□	■
Deuterostomia	■	■	■	■	■	■	■	■	□	■

Black square represents presence, while white square represents absence.

In addition to these eight ancestral eukaryotic C1A lineages, two orthologous gene families exist in Obazoa. The first is the well-known cathepsin O, which is widespread in metazoans and present throughout opisthokonts (Choanoflagellata, Filasterea, Tunicaraptor, Pluriformea, and Ichthyiosporea). Similar to other ancestral eukaryotic C1A lineages, cathepsin O is lost in Fungi and Rotosphaerida (Table 2). It is also present in Apusozoa but absent from Breviatea. A new, longer C1A peptidase that contains a vWFA domain (vWFA-C1 peptidase) is present in metazoans, and while it is quite widespread in basal metazoans and Protostomia, it is very rare in Deuterostomia (found only in some echinoderms and a few fishes) (Table 2). In addition to Metazoa, it can be found only in Fungi and Choanoflagellata. This peptidase has a sparse distribution in other eukaryotic lineages and can be found only in a few isolated cases, namely, in green algae, Cryptista, Centroheliozoa, the SAR supergroup, and Discoba. In these lineages, vWFA-C1 peptidases are present in only one or two species; thus, it is most likely that they were horizontally acquired.

#### 2.3.2. Papain Family in Prokaryotes

The analysis of the papain family distribution in Bacteria showed its widespread presence in all the major phyla. The only exception is the phylum Dyctioglomi, which has genome data for only two species and shows the absence of the papain family (Table 3). Similarly, the papain family is widespread in Archaea and is present in all the major archaeal lineages (Table 3). 

**Table 3:** Papain superfamily is widespread in Bacteria and Archaea.

Taxonomic Group/Phylum	Single Domain C1A Peptidases	Multidomain C1A Peptidases
**Bacteria**	●	●
Acidobacteria	●	●
Aquificae	●	●
Atribacterota	●	●
Caldiserica/Cryosericota group	●	●
Calditrichaeota	●	●
Chrysiogenetes	●	●
Coprothermobacterota	●	●
Deferribacteres	●	●
Desulfobacterota	●	●
Dictyoglomi	○	○
Elusimicrobia	●	●
FCB group	●	●
-Bacteroidetes/Chlorobi group	●	●
-Fibrobacteres	●	●
-Gemmatimonadetes	●	●
Fusobacteria	●	●
Myxococcota	●	●
Nitrospinae/Tectomicrobia group	●	●
Nitrospirae	●	●
Pseudomonadota (=Proteobacteria)	●	●
PVC group	●	●
-Chlamydiae	●	●
-Lentisphaerae	●	●
-Planctomycetota	●	●
-Verrucomicrobia	●	●
Spirochaetes	●	●
Synergistetes	●	●
Terrabacteria group	●	●
-Actinomycetota	●	●
-Bacillota (=Firmicutes)	●	●
-Chloroflexi	●	●
-Cyanobacteria/Melainabacteria group	●	●
-Deinococcus-Thermus	●	●
-Tenericutes	●	●
Thermodesulfobacteria	●	●
Thermotogae	●	●
Bacteria candidate phyla	●	●
**Archaea**	●	●
Asgard group	●	●
Thermoplasmatota	●	●
DPANN group	●	●
Euryarchaeota	●	●
TACK group	●	●

Black circle represents presence, while white circle represents absence.

The number of C1A peptidases in prokaryotic genomes is very low; they are mostly present as a single sequence, although occasionally, up to three sequences may also exist. In some species, only complex multidomain proteins with a C1 domain are present, whereas others possess short C1A peptidases, which, from an evolutionary point of view, are more important as progenitors of eukaryotic C1A peptidases. Although numerous prokaryotic C1A peptidases are present in the form of complex multidomain proteins, they were not analysed in more detail here because they cannot be potential progenitors of eukaryotic C1A peptidases. Short C1A peptidases are widespread in both Bacteria and Archaea (Table 3). However, a simple presence/absence pattern can be misleading as horizontal gene transfer is quite common between different bacteria and between Bacteria and Archaea [52,53]. Often, a spotty distribution of C1A peptidases in large bacterial phyla is observed. As evident from the MEROPS Db, the distribution of C1A peptidases in Bacteria is not uniform, and often, only a single species possesses a C1A peptidase. However, the analysis of a large collection of bacterial genomes often shows a different situation from that of the MEROPS Db data since genome data covers a much larger taxonomic diversity than the MEROPS Db. Moreover, a brief analysis of the signal peptides in prokaryotic C1A peptidases showed that they are present in extracellular proteins but absent in intracellular proteins. 

### 2.4. Diverse Evolutionary Forces Are Reshaping the Papain Family

The evolution of C1A peptidases (Table 4) is driven by several forces. The major players are gene duplication, horizontal gene transfer, and gene loss.

#### 2.4.1. Gene Duplication

Gene duplication within the papain family is almost restricted to eukaryotes, as only a few cases can be found in prokaryotes. It is well known that gene duplication in prokaryotes is not as common as in eukaryotes and that the major force for prokaryotic adaptation is the acquisition of novel genes by horizontal gene transfer [52,53]. Numerous gene duplications in the eukaryotic ancestor resulted in the emergence of the eight ancestral eukaryotic C1A lineages. A number of bursts in functional diversification, as evidenced by large lineage-specific expansions resulting in large multigene families, have occurred mostly in the cathepsin L orthologous gene family in unicellular eukaryotes, land plants, invertebrates, and some lineages of placental mammals (e.g., placental cathepsins in rodents) [54,55,56,57]. In eukaryotes, the cathepsin B orthologous gene family has remained either as a single gene or as small multigene families that sometimes undergo bursts in functional diversification, such as in aphids [58] or in plants [59]. In contrast to cathepsin B, the cathepsin L orthologous gene family has experienced more complex and dynamic evolution through numerous gene duplications, the majority of which are species-specific. The consequence of these large lineage-specific expansions is that cathepsins L are the most numerous representatives of the papain family. In two rotifer species, subsequent gene duplications of horizontally acquired genes from cyanobacteria generated large lineage-specific expansions of C1A peptidases, from tens to over a hundred sequences per species. Additionally, evolutionarily younger C1A orthologous gene families originated in vertebrates (cathepsins W, K, S, and L2) by gene duplications [60]. 

#### 2.4.2. Horizontal Gene Transfer 

Horizontal gene transfer (HGT) is a very common process in prokaryotes [52,53,61,62,63]; thus, it is not surprising that HGT is well represented in the prokaryotic part of the papain family. Many cases of horizontally acquired C1A peptidases can be observed in prokaryotes, and the most obvious cases are present in Archaea. The easiest way to recognise HGT is with homology searching. Unusually high levels of sequence conservation between distantly related organisms are a clear indication of HGT [52,53]. In prokaryotes, HGT occurs in two directions: between diverse bacterial taxa and between Bacteria and Archaea. We found that Archaea possess 44% Archaea-specific short C1A peptidases, while 56% are acquired through HGT from Bacteria (Supplementary File S1). In Archaea, we found that the majority of the HGT cases of short C1A peptidases are concentrated in methanogenic Archaea (in Euryarchaeota—in the Stenosarchaea and Methanomada groups). Surprisingly, in the evolutionarily very important Asgardarchaeota (closest relatives of eukaryotes) [64], we found that the vast majority of short C1A peptidases are horizontally acquired from Bacteria. Based on our numerous homology searches between diverse bacterial taxa, we found that the extent of HGT within Bacteria is similar to that observed in Archaea, although Bacteria possess more short C1A peptidases. The biological reason for such a high level of HGT in Archaea and Bacteria is related to the ecology of these organisms, as they prevail in microbial mats and biofilms where HGT is very common among diverse taxonomic lineages of microbes [65].

We found at least five separate and independent cases of HGT of C1A peptidases from diverse bacteria to eukaryotes (Supplementary File S2). The first case is a recent HGT from cyanobacteria to two rotifer species. The second case is the HGT of short bacterial C1-terB peptidases to the Ascomycota fungi. The third case is the HGT of long bacterial C1A peptidases to a few green algae and two chytrid fungi. The fourth case is the HGT of a long C1A peptidase from Streptomycetes to Ascomycota fungi. The fifth case involves the HGT of short C1A peptidases from bacteria to the SAR supergroup (mostly dinoflagellates). In contrast to the above-described HGT cases, eukaryotes have very few cases of HGT between different eukaryotic lineages. The most obvious case occurs in plant fungal pathogens, where diverse plant cathepsins are horizontally acquired from plant hosts (Supplementary File S2).

We also found several examples of HGT of C1A peptidases from diverse eukaryotes to DNA viruses, as well as from bacteria to DNA viruses. In the case of HGT of C1A peptidases from eukaryotes to DNA viruses, lepidopteran cathepsin F (also called V-cath peptidase) was acquired by nucleopolyhedroviruses and granuloviruses (Baculoviridae). Cathepsin B was independently acquired horizontally by lymphocystisviruses (Iridoviridae), ascoviruses (Ascoviridae), and algal phaeoviruses (Phycodnaviridae). Short bacterial C1A peptidases (xylellain-type) were independently horizontally acquired by very large DNA viruses (Mimiviridae, Megaviricetes) and by Myoviridae and Siphoviridae (Caudoviricetes) (Supplementary File S2). Most of these HGT cases can also be found in the MEROPS Db, although the genome databases show a larger number of cases.

#### 2.4.3. Gene Loss

Gene loss within eukaryotes can be easily recognised since we inferred the ancestral state of the papain family from the presence of eight ancestral eukaryotic C1A peptidases within the same genome. We demonstrated that diverse ancestral C1A peptidases have been lost several times in numerous eukaryotic genomes or in entire taxonomic groups (Table 1 and Table 2). We also inferred ancestral states for all eukaryotic supergroups (Table 5). Demonstrating the ancestral state of the papain family is important for the recognition of several independent cases of gene loss in diverse orthologous gene families in distinct eukaryotic taxonomic groups. Some taxonomic groups with very large genome data coverage, such as fungi, lost all ancestral eukaryotic C1A peptidases. The evidence for these C1A losses is based on the analysis of the complete genomes. Gene loss in the papain family can cause problems in the correct interpretation of evolutionary history. However, the large sampling of diversity in the papain family throughout eukaryotes and prokaryotes enabled us to correctly interpret their evolutionary history.

### 2.5. Origin and Early Diversification of the Papain Family

From the distribution pattern of C1A peptidases in Archaea and Bacteria, we can infer that they were present in the last universal common ancestor (LUCA) [66] (Table 6). However, HGT may present a problem when inferring the origin of the papain family. However, in Archaea, we found that 44% of the short C1A peptidases were Archaea-specific, but many more unique Bacteria-specific C1A peptidases were present in Bacteria. Thus, despite the vast amount of HGT in prokaryotes, we found a sufficient number of short C1A peptidases in Archaea and Bacteria that support their presence in LUCA. The ancestor of the papain family is related to the short prokaryotic C1A peptidases.

The analysis of the presence of C1A peptidases in Asgardarchaeota, which are assumed to be the closest relatives of eukaryotes [64], revealed the presence of several representatives; however, most were horizontally acquired from diverse bacterial taxa. We also found that short C1A peptidases are quite rare in diverse Alphaproteobacteria, especially in Rickettsiales, which are assumed to be the progenitors of mitochondria. In the progenitors of chloroplasts, the Cyanobacteria, we identified a diverse collection of short C1A peptidases. In the syntrophy model of eukaryogenesis [67], Deltaproteobacteria is proposed as an additional taxon involved in eukaryogenesis, together with an archaeal host and a mitochondrial ancestor. We found that Deltaproteobacteria have a diverse collection of short C1A peptidases. Regarding the problems associated with the abundance of HGT events among prokaryotes and the rarity of C1A peptidases in the key taxa important for eukaryogenesis (Asgardarchaeota and Alphaproteobacteria), we may infer that in the first eukaryotic common ancestor (FECA), C1A peptidases may also have been acquired through HGT from some bacterial taxa. In the case of the tripartite syntrophy model, C1A peptidase could have been obtained from Deltaproteobacteria. Owing to the intense HGT in Asgardarchaeota [64], we believe that the C1A peptidase was horizontally acquired from bacteria (either in the bacterial symbiont or in the archaeal host). Therefore, the ancestor of eukaryotic C1A peptidases was most likely of bacterial origin. Regardless of the origin of the prokaryotic C1A peptidase in FECA (archaea-specific, bacteria-specific, or horizontally acquired bacterial C1A), there is no doubt that only a single ancestor of eukaryotic C1A peptidases existed in FECA. During the long transition period from FECA to LECA [68], additional C1A peptidases may have been horizontally acquired from diverse bacterial taxa. During the transition period between FECA and LECA, intensive reshaping of the C1A repertoire occurred. While eight ancestral C1A lineages were present in LECA, only a single C1A ancestor was present in FECA. Regarding the differences among ancestral C1A paralogs, we can infer a simple origin for the four ancestral C1A lineages that possess the I29 domain. From their progenitor, a series of gene duplications was responsible for the emergence of cathepsins L, F, H, and 26/29 kDa peptidases. In the case of cathepsins B, C, and X, there was also a large structural sequence divergence among them; therefore, we cannot exclude the possibility of their origin through separate HGT events. However, an alternative possibility, which is also supported by the phylogenetic analysis (Figure 1), is that they originated through a few gene duplications. In this case, diverse propeptides were acquired separately (e.g., the exclusion domain in cathepsin C). In the case of cathepsin B, it was evident that some eukaryotic lineages may possess simpler forms without the propeptide domain, which can also be a consequence of the higher sequence divergence that prevents the recognition of this protein domain. Thus, during the transition period from FECA to LECA, many C1A lineages originated, some of which may have been lost. Surprisingly, prokaryotes have few C1A peptidases per species that are present either as single-domain C1As or complex multidomain proteins. From the ancestral eukaryotic C1A repertoire, it is evident that intensive functional diversification is responsible for generating such a large diversity in the papain family in the LECA.

### 2.6. Structure–Function Relationships in the Papain Family

**Figure 3:**
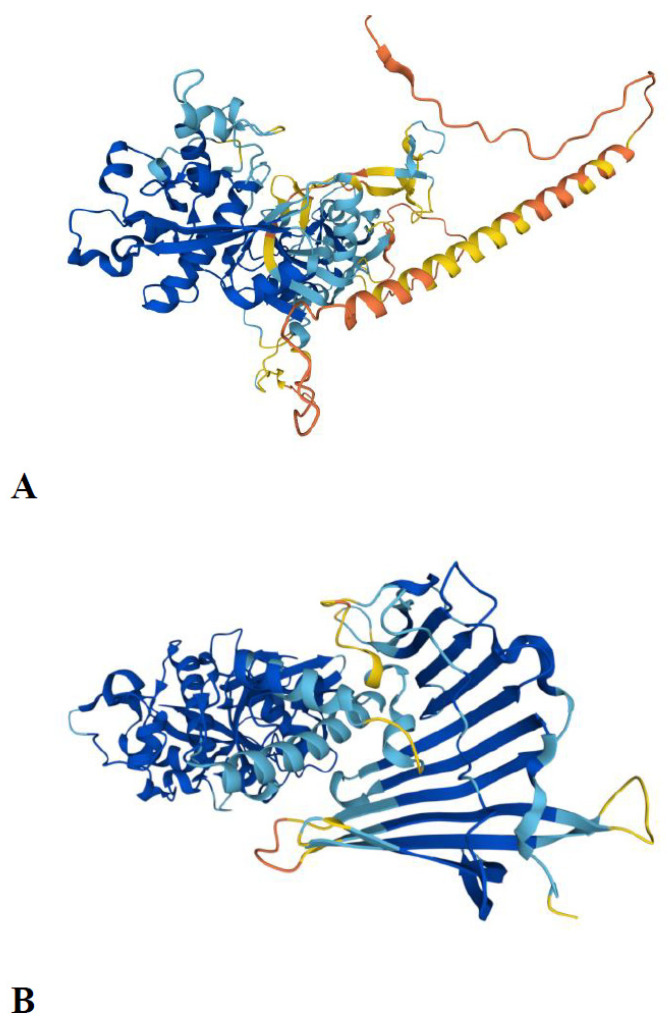
AlphaFold-predicted structures of the two ancestral C1A lineages. (**A**) AlphaFold-predicted structure of the type 1 long C1 peptidase from *Phytophthora infestans* (A0A833SVU0_PHYIN). The transmembrane domain is located at positions 32–53. (**B**) AlphaFold-predicted structure of the 26/29 kDa peptidase from *Aedes albopictus* (midgut cysteine proteinase 2; A0A182HAH0_AEDAL).

The discovery of eight ancestral paralogous C1A lineages is of utmost importance for the recognition of the structure–function relationships within the papain family. Since we know what the ancestral eukaryotic C1A lineages are, we can also use the known data on their functions to make some generalisations. Most of these eukaryotic ancestral C1A lineages have been functionally and biochemically studied in metazoans (including parasites), plants, and protists (mostly in diverse pathogens) [3,4,5,6]. Although they have different functions in diverse eukaryotic lineages, these functions are related to lifestyle. In most cases, C1A peptidases are involved in protein degradation, regulated proteolysis, and largely participate in the host–pathogen arms race. In the latter case, they are either used in attack (C1A peptidases of bacteria and eukaryotic pathogens and parasites) or defence (in multicellular eukaryotes), where the papain family represents an important part of the innate immune system [69]. Most of the ancestral C1A lineages have been functionally and biochemically characterised; the only exception is the type 1 long C1 peptidase, for which no such data exist. However, genomic data showed that these peptidases are present in the form of large multigene families (Figure 2). Since they are common in protist pathogens, we can infer that they probably play an important role in the host–pathogen arms race, most likely in host invasion. Additionally, the functions of bacterial ancestral-type short C1A peptidases are still scarce and limited to two species [70,71]. Three-dimensional structures are available for six eukaryotic ancestral paralogous C1A lineages, for cathepsins B [18], C [25], X [23], L/papain [17,20], F [27], and H [22], while no structures are available for the “insect 26/29 kDa peptidase” or the “type 1 long C1 peptidase”, although high-quality models can be obtained with AlphaFold [72] (Figure 3). The AlphaFold-predicted structures for both ancestral C1A lineages highlight the conservation of the papain-like fold domain in both structures (labelled with blue colour), whereas several segments that were suboptimally predicted (labelled with yellow/orange colour) correspond to additional domains with very low homology of these multidomain proteins, namely, the growth factor receptor domain family (Figure 3A) and the LolA fold (Figure 3B), respectively. Very recently, a LolA/EPDR domain was found in the N-terminal part of the 26/29 kDa peptidases [73]. These peptidases were formed during eukaryogenesis by the fusion of a LolA/EPDR protein with the ancestral cathepsin L. The LolA fold is a barrel-like fold comprising an 11-stranded antiparallel β-sheet with a short helix located within its centre. Although the hydrophobic cavity of the LolA fold represents a possible binding site for the lipid moiety of lipoproteins, its role in the 26/29 kDa peptidases might be different.

Discovering the repertoire of ancestral eukaryotic C1A peptidases is important for directing structural studies of the papain family, as its structural diversity in eukaryotes is already well-known. Even in prokaryotes, two 3D structures are available for single-domain (or short) C1A peptidases [70,71], and one structure is available for multidomain C1A peptidase, where the lectin domain is fused with the C1A domain [74]. For evolutionarily younger eukaryotic C1A lineages (e.g., cathepsin O and vWFA-C1), high-quality models can be easily produced using AlphaFold [72]. Crystal structures of the diverse eukaryotic [17,18,20,21,22,23,25,27] and short prokaryotic [70,71] C1A peptidases have shown that their core catalytic machinery and fold is highly conserved throughout evolution. Thus, structure–function relationships are known for the majority of eukaryotic ancestral C1A peptidase lineages. It is also important to solve these issues for less-studied C1As or the new C1A ancestral lineage that has not yet been analysed from a structure–function point of view. Thus, the structure–function knowledge of the papain family is in its “ripe form”, as it is more or less completely solved. 

## 3. Materials and Methods

### 3.1. Data Mining

All database searches were performed online and were completed in January 2023. The databases analysed were the nonredundant (NR), TSA, WGS, and microbial and eukaryotic genome databases of the NCBI (http://www.ncbi.nlm.nih.gov (last accessed on 30 January 2023)). Diverse taxon-specific eukaryotic and prokaryotic proteome, transcriptome, and genome databases were searched using the NCBI website. Attempts were made to identify novel representatives of the papain family in diverse proteomic databases, such as MEROPS (merops.sanger.ac.uk (last accessed on 30 January 2023)) [1], Superfamily (supfam.org (last accessed on 30 January 2023)), and InterPro (www.ebi.ac.uk/interpro/ (last accessed on 30 January 2023)). Crucial eukaryotic taxa and lineages not available in the NCBI databases were searched for C1A peptidases in the EukProt V3 transcriptome database (https://evocellbio.com/eukprot/ (last accessed on 30 January 2023)) [50]. To identify all the available representatives of the papain family, database searches were performed iteratively. Comparisons were performed using the TBLASTN and BLASTP tools [75], with an E-value cut-off set to 10^−5^ and default settings for the other parameters. Diverse eukaryotic (e.g., human, invertebrate, and eukaryotic pathogens), prokaryotic (bacterial and archaeal), and viral C1A peptidases were used as queries. The C1 domain in newly discovered representatives of the papain family was identified using the NCBI CDD domain database (https://www.ncbi.nlm.nih.gov/Structure/cdd/wrpsb.cgi (last accessed on 30 January 2023)). The Translate program (http://www.expasy.org/tools/dna.html (last accessed on 30 January 2023)) was used to translate DNA sequences.

### 3.2. Phylogenetic Analysis

Due to the high number of sequences available from the papain family, only representatives of the C1A peptidases from eukaryotic supergroups and a few prokaryotic representatives of the papain family were included in the analyses. In this way, the stable backbone phylogeny was obtained. Protein sequences were aligned using Clustal Omega [76]. Phylogenetic trees were reconstructed using the ML method [77]. The reliability of the resulting topologies was evaluated using 1000 bootstrap replications. Diverse prokaryotic representatives of the papain family were used as an outgroup. Phylogenetic analyses were performed using the program IqTree [77], whereas tree visualisation was performed using iTOL v6 [78].

## 4. Conclusions

We performed a comprehensive phylogenomic analysis of the papain family, using extensive proteomic, transcriptomic, and genomic data from the Archaea, Bacteria, and Eukaryota, and obtained new insights into the origin and evolution of C1A peptidases. In contrast to the widely accepted view that eukaryotes possess only two ancestral C1A lineages, the cathepsin B and L classes [45], our study shows that such a view is limited. Here, we demonstrated that eight ancestral eukaryotic paralogous C1A peptidase lineages were present in the ancestor of eukaryotes. These eight ancestral eukaryotic C1A peptidase lineages are cathepsins B, C, X, L, H, and F, 26/29 kDa peptidase, and type 1 long C1 peptidase, which are present in all eukaryotic supergroups. The key findings of our study report that the papain family was present in the LUCA and that this family was already highly diversified in the LECA. Altogether, our study provides an in-depth understanding of the diversity and evolution of the papain family.

## Figures and Tables

**Table 4:** Evolutionary forces acting on the C1A peptidases.

Evolutionary Forces	Archaea	Bacteria	Eukaryota	Viruses
Gene duplication	○	○	●	○
Gene loss	●	●	●	●
Horizontal gene transfer	●	●	●	●
Functional diversification	○	○	●	○
Gene/domain fusion	●	●	●	○
C1 domain duplication	○	○	●	○
Loss of active site	○	○	●	○
Change of the peptidase class	○	○	●	○
Lineage-specific expansion	○	○	●	○
Coevolution of peptidases with their inhibitors (arms race)	●	●	●	●
Alternative splicing	○	○	●	○
Pseudogenization	○	○	●	○
Expression divergence	○	○	●	○

Black circle represents presence, while white circle represents absence.

**Table 5:** Ancestral states for the eight ancestral eukaryotic paralogous C1A peptidases in major eukaryotic supergroups.

	Cath B	Cath C	Cath X	Cath L	Cath F	Cath H	26/29 kDa Peptidase	Type 1 C1 Long
**Diaphoretickes**	■	■	■	■	■	■	■	■
**Amorphea**	■	■	■	■	■	■	■	■
CRuMs	■	■	■	■	■	■	□	□
Ancyromonadida	■	■	■	■	■	□	■	□
Malawimonadida	■	■	■	■	■	□	■	□
**Discoba**	■	■	■	■	■	■	■	■
**Metamonada**	■	■	■	■	■	□	■	■
**LECA**	■	■	■	■	■	■	■	■

Black square represents presence, while white square represents absence.

**Table 6:** C1A peptidases that were present in LUCA, FECA, LECA and in the key prokaryotic lineages.

	C1A	Cath B	Cath C	Cath X	Cath L	Cath F	Cath H	26/29 kDa Peptidase	Type 1 C1 Long
**LECA**	□	■	■	■	■	■	■	■	■
**FECA**	■	□	□	□	□	□	□	□	□
**α-Proteobacteria**	■	□	□	□	□	□	□	□	□
**Cyanobacteria**	■	□	□	□	□	□	□	□	□
**δ-Proteobacteria**	■	□	□	□	□	□	□	□	□
**Archaea**	■	□	□	□	□	□	□	□	□
**LUCA**	■	□	□	□	□	□	□	□	□

Black square represents presence, while white square represents absence.

## Data Availability

The data presented in this study are available in the article and supplementary material. All new sequence data for the papain family will be sent to the MEROPS database.

## Supplementary Materials

The following supporting information can be downloaded at: https://www.mdpi.com/article/10.3390/ijms241411761/s1.

## Abbreviations

FECA: first eukaryotic common ancestor; HGT, horizontal gene transfer; LECA, last eukaryotic common ancestor; LUCA, last universal common ancestor; Db, database; cath, cathepsin.
