# Supplementary material for: Origin and Early Diversification of the Papain Family of Cysteine Peptidases
Source: Int J Mol Sci. 2023 Jul 21;24(14):11761. doi: 10.3390/ijms241411761 (PMC10380794; doi:10.3390/ijms241411761)
Supplement: Supplementary file 1 [file ijms-24-11761-s001.zip › ijms-2491259-supplementary.pdf]

Supplementary File S1 Archaeal short C1A peptidases

Archaea-specific short C1A peptidases

DAHWUS010000703, UCB59168, MCK4996027, MCK5636955, RLF37898, MCK4365330, MCD6513258, KAA0002332, MCI4345830, MCI4341662, MCI4334896, MBE3121439, HHF56427, MCD6216108, MCF7862147, PKP61290, NCN64603, MBU1246324, MCK5627663, MBL7168114, RLG75435, RLG02684, NLE04568, RLF09379, KAB3546236, RZN40145, RLG25020, MCK4459896, PXF57508, MCK5108675, MCE7699474, MCE7699050, MBS7626718, MBS7650961, HIH98053, KYC53855, MBS7646162, MBC7109069, HIH98781, MBS7622855, HIH29766, WP_148687067, VVB58332, MCE7699047, RLI87757, MBQ6628014, RLF92273, VVB65043, MBP7069777, KXB08553, VVB93671, HEC72648, MBP1662758, MCI4337786, VVB62775, MCK5561106, MCK9567651, KYK35576, MCK4902719, MBS3749108, OYV09165, MBS3801904, MCI4363036, NTV76584, OYV14351, KYK28083, MCJ7697462, HIH28190, HIH00289, OYT28409, NYT01874, MBC7081689, MBU0496552, MBC7128408, KYK22496, RLF39497, DAC73334, MBN1861190, RLF51075, MBY8998716

Horizontally acquired short C1A peptidases in Archaea

MBD3407302, MCK5614944, MCJ7698565, MCK4415586, RLF27795, UCE36941, MBW2982642, MBN2335896, MCH8905524, TFH41208, MBM4250075, OIO22098, VVB93824, MCE8423104, VVB85898, OIO24549, VVC01043, MCK9586153, MBP7067802, NLJ21726, UEC41668, OPX81696, MCK9405279, WP_013720700, NMB85868, OPX75105, MCK4347891, MBK7387220, WP_209584139, WP_071907541, OPY48778, WP_209631153, WP_219968434, PWR71151, WP_012901135, RLG13091, OPY19415, WP_174590212, MCG2737956, WP_014406686, RMF28885, MBN1678530, OLD12351, NLV28195, CAD7778915, OPY18197, WP_013825867, CAD7776905, MBP8624268, MCP8310751, WP_069582411, NLH22408, WP_048082145, MCJ7464255, MCE5215344, TMI41914, MRR14384, MCP8322871, NTV76414, KYK32179, MCJ7445856, OYV10600, TMI69218, TFH41693, OLE91984, OQB17203, OPY54508, MCK9597148, MCI4435550, MCP8304908, MPZ06446, QNO58023, TRZ67270, QQG48186, WP_134482916, VFJ12905, MCK4734469, MBN1324555, WP_248535176, MCK4971339, OPY55129, CAD7780064, MBU0762495, TRZ69458, WP_048182473, HIH19026, HDL02487, RLG20759, RLG35358, WP_095644254, WP_048153359, HII92371, WP_229395323, WP_048117302, QNO57621, MCH8905524, RLI65907, MCK4266327, WP_048180826, NLH20743, OYV14776, VVB88503, KYK28850,

Supplementary File S2 Horizontally acquired C1A peptidases in eukaryotes and viruses

Only the most representative sequences are listed. Directions of horizontal transfer:

A) Bacteria to Eukaryota

A1) very recent HGT from Cyanobacteria to bdelloid rotifers (Bdelloidea, Rotifera)

donor sequence: WP_054465464
bdelloid rotifers: Rotaria (CAF3488612), Adineta (CAF1251741), Didymodactylos (CAF1112757)

A2) Streptomycetes to Fungi

donor sequence: WP_073773949
Fungi: KAF2749223, KAI1849739, XP_003656030, KAH6627468

A3) Gammaproteobacteria to green algae and chytrid fungi

donor sequence: WP_242518642
green algae: KAI9000141, KAG2487069, PNH11230, XP_042927188, XP_002956830
chytrid fungi: KAI9024262

A4) Deltaproteobacteria to Fungi (Ascomycetes) and diatoms - C1-terB sequences

donor sequence: WP_136935198
Fungi (Ascomycetes): TLD34137, KLU91290
diatoms: CAB9506488

A5) Cyanobacteria to dinoflagellates and cryptophytes (Alveolata, SAR supergroup) - C1-EFh sequences

donor sequence: WP_171575278
dinoflagellates: CAE7556063, ICPI01040318, GICE01035329
cryptophytes: XP_005833280

A6) Actinobacteria to pelagophytes (SAR supergroup, Stramenopiles; Ochrophyta; Pelagophyceae) - pelagophytes causes harmful marine brown tide blooms

donor sequence: TMM39349
pelagophytes: KAH8058674, KAJ1448925

B) Bacteria to DNA viruses (Caudoviricetes)

Donor sequence: WP_249293808, PWM09368, WP_230141817, MCI6929133
Caudoviricetes: DAJ83460, DAT91692, DAI08572, DAH93260, DAR43207, DAM15906

C) plants to fungal plant pathogens – evidence from the fungal genome sequences

Triticum aleurain: Pyricularia oryzae (MQQU01001077)
RD21A-like (*Juglans regia*): Xylaria sp. (JADKYC010001065)
